# Positive urinary fluorescence *in situ* hybridization indicates poor prognosis in patients with upper tract urothelial carcinoma

**DOI:** 10.18632/oncotarget.24007

**Published:** 2018-01-04

**Authors:** Bao Guan, Yicong Du, Xiaohong Su, Zhenpeng Cao, Yifan Li, Yonghao Zhan, Ding Peng, Gengyan Xiong, Dong Fang, Yi Ding, Shiming He, Yanqing Gong, Qun He, Xuesong Li, Liqun Zhou

**Affiliations:** ^1^ Department of Urology, Peking University First Hospital, Xicheng, Beijing, China; ^2^ Institute of Urology, Peking University, National Urological Cancer Center, Beijing, China

**Keywords:** fuorescence in situ hybridization (FISH), upper tract urothelial carcinoma (UTUC), recurrence, prognosis

## Abstract

Here, we evaluated the potential contribution of fluorescent in situ hybridization (FISH) as a prognostic risk factor of bladder recurrence and survival in patients with upper tract urothelial carcinoma (UTUC). A total of 159 UTUC patients were enrolled in this study from January 2012 to May 2016. The 159 voided urine samples before surgery were analyzed using the UroVysion^®^ kit to detect the copy numbers of chromosomes 3, 7, 17 and 9p21 (p16). Patients were classified using an optimal cutoff value of chromosomes 3, 7, 17, and 9p21. Cox's proportional hazards regression model was used to assess the prognostic value of FISH for bladder recurrence and survival. We found that 27 (17.6%) patients experienced bladder recurrence and 26 (16.4%) patients died from cancer, with a median follow-up of 27 months. The patients with positive FISH result were more likely to present bladder recurrence (*p* = 0.077). However, positive FISH was not associated with cancer specific-free survival (CSS) (*p* = 0.944). Tumor multifocality, the percentage of abnormal chromosome 3 > 5%, chromosome 7 > 6%, chromosome 17 > 11% and deletion of p16 > 4% were significant prognostic risk factors for BRFS in univariate analysis. In multivariate analysis, only tumor multifocality (hazard ratio [HR] = 3.487, 95%CI: 1.605–7.576, *p* = 0.002) and the percentage of p16 loss > 4% were both prognostic risk factors for bladder recurrence (HR = 3.487, 95%CI: 1.605–7.576, *p* = 0.002). These data consider that the urinary FISH test could be a powerful tool in predicting the risk of bladder recurrence in patients with UTUC.

## INTRODUCTION

Upper tract urothelial carcinoma (UTUC) is a relatively uncommon malignant tumor [1]. Though the standard treatment method, radical nephroureterectomy (RNU), could remove primary tumor lesions, the probability of bladder recurrence and progression remain high [1–3]. Consequently, it is necessary to find novel biomarkers to predict oncological outcomes.

Fluorescence *in situ* hybridization (FISH) analysis which uses nucleic acid probes marked with fluorescence to evaluate cells in the voided urine for chromosomal alterations and be widely applied to the field of cancer diagnosis. [4–7], and we had identified its high sensibility and prediction for advanced UTUC [8]. We used UroVysion^®^ FISH test ((Abbott Molecular, des Plaines, IL, USA)) to measure the percentage of particular copy number variation of chromosomes 3, 7, 17, and 9p21 (p16) in the urine. Recently, several studies had reported that FISH could be used as an aid to predict prognosis in bladder cancer patients [9–11], but its prognostic value had not yet been confirmed in UTUC patients. Therefore, the prognostic capacity of FISH was studied by us in a single and relatively high-volume center in China.

## RESULTS

### Patient and tumor characteristics

There were 78 (49.1%) male and 81 (50.9%) female patients, and the median age was 70 years (range: 31–87 years). Among 159 patients, non-muscle invasive (≤ pT1) and muscle invasive (≥ pT2) UTUC patients were 82 (51.5%) and 77 (48.5%), respectively, while low and high grade patients were 86 (54.1%)and 73 (45.9%), respectively (Table 1).

**Table 1:** Characteristics and outcomes of UTUC patients

Variables	N (%) or Median (range)
N(%)	159
Gender	
Male	78(49.1)
Female	81(50.9)
Age	70(31-87)
Hydronephrosis	
Present	19(11.9)
Absent	140(88.1)
Tumor location	
Pelvis	81(50.9)
Ureter	71(44.7)
Both	7(4.4)
Tumor stage	
A	12(7.5)
1	70(44.0)
2	39(24.5)
3	37(23.3)
4	1(0.6)
Tumor grade	
Low	86(54.1)
High	73(45.9)
Lymph node status	
cN0 or pN0	152(95.6)
N+	7(4.4)
LVI	
Presence	139(87.4)
Absence	20(12.6)
Tumor size	
≤3cm	90(56.6)
>3cm	69(43.4)
Multifocal	
Yes	136(85.5)
No	23(14.5)
FISH	
Positive	102(64.2)
Negative	57(35.8)
FISH probe	
CH3 abnormal, %	7(0-46)
CH7 abnormal, %	7(0-38)
CH17 abnormal, %	8(0-53)
p16 loss, %	7(0-65)
Outcome	
Follow-up, months	27(3-55)
Time to recurrence, months	11(1-43)
Time to death, months	15.5(3-38)

LVI = lymphovascular invasion; FISH = fluorescence in situ hybridization; CH = chromosome.

### FISH results

Clinical outcomes and cutoff value are described in Table 2. The median percentage of abnormal chromosome 3, 7, 17 and p16 loss were 7% (0–46%), 7% (0–38%), 8% (0–53%) and 7% (0–65%), respectively. According to the area under the curve (AUC) from the receiver operating characteristic curve (ROC) for intravesical relapse, we defined the cutoff values of the proportion of aberrant chromosomes 3, 7, and 17 and loss of p16. The cutoff values were 5% for chromosome 3, 6% for chromosome 7, 11% for chromosome 17 and 4% for p16 loss. According to the cutoff values of preoperative factors, all patients were allocated to 2 classes.

**Table 2:** Clinical outcome in 159 assessable patients during median follow-up of 28 months

FISH probe	N(%)	Cancer specific death	Recurrence
FISH			
Positive	102(64.2%)	16(15.7%)	22(21.6%)
Negative	57(35.8%)	10(17.5%)	6(10.5%)
CH3 abnormal			
≤ 5%	62(39.0)	11(17.7%)	5(8.1%)
> 5%	97(61.0)	15(15.5%)	23(23.7%)
CH7 abnormal			
≤ 6%	77(48.4)	15(19.5%)	9(11.7%)
> 6%	82(51.6)	11(13.4%)	19(23.2%)
CH17 abnormal			
≤ 11%	98(61.6)	15(15.3%)	13(13.3%)
> 11%	61(38.4)	11(18.0%)	15(24.6%)
p16 loss			
≤ 4%	60(37.7)	6(10.0%)	4(6.7%)
> 4%	99(62.3)	20(20.2%)	24(24.2%)

FISH: fluorescence in situ hybridization; CH: chromosome.

The association of FISH with pathological tumor characteristic is shown in Table 3. Patients with percentage of chromosome 17 > 11% and loss of p16 > 4% were associated with high stage and grade. Univariate and multivariate binary logistic analysis found only the percentage of abnormal chromosome 17 was associated with invasive UTUC (hazard ratio [HR] = 3.027, 95%CI: 1.059–8.650, *p* = 0.015). Other preoperative factors did not show statistically significant differences.

**Table 3:** Preoperative factors that predict high-risk UTUC in univariate and multivariate analyses

Preoperative factors	T2-T4				Tumor high grade			
	Univariate		Multivariate		Univariate		Multivariate	
	X^2^	*P*	HR (95% CI)	*P*	X^2^	*P*	HR (95% CI)	*P*
Gender (male vs female)	2.752	0.097	1.483(0.748–2.938)	0.259	0.143	0.705	0.956(0.483–1.892)	0.897
Age (≤ 70 vs > 70)	1.388	0.239	1.562(0.797–3.063)	0.194	2.191	0.139	1.696(0.869–3.310)	0.122
Hydronephrosis (absence vs absence)	1.875	0.171	0.517(0.479–4.322)	0.517	0.018	0.892	1.283(0.442–3.724)	0.647
Tumor location (both or ureter vs pelvis)	1.239	0.538	1.679(0.392–1.366)	0.327	3.915	0.141	1.518(0.820–2.813)	0.184
Tumor size (≤ 3 cm vs > 3 cm)	2.155	0.142	1.679(0.836–3.372)	0.146	0.180	0.671	1.060(0.530–2.119)	0.869
Multifocal (presence vs absence)	0.705	0.401	1.167(0.451–3.018)	0.751	1.219	0.270	1.529(0.592–3.950)	0.381
CH3 abnormal (≤ 5% vs > 5%)	0.969	0.325	0.802(0.282–2.284)	0.680	3.180	0.075	1.099(0.392–3.081)	0.858
CH7 abnormal (≤ 6% vs > 6%)	1.855	0.173	0.584(0.179–1.906)	0.373	2.905	0.088	0.674(0.210–2.160)	0.506
CH17 abnormal (≤ 11% vs > 11%)	5.925	**0.015**	3.027(1.059–8.650)	**0.039**	3.848	**0.050**	1.436(0.518–3.979)	0.487
p16 loss (≤ 4% vs > 4%)	3.932	**0.047**	1.762(0.709–4.381)	0.223	7.875	**0.005**	2.279(0.918–5.658)	0.076

HR = hazard ratio; CI = confidence interval; CH: chromosome.

### Oncological outcomes

The median follow-up was 27 months (range: 3–55). The median time to bladder relapse and time to death from UTUC was 11 (range: 1–43 months) months and 15.5 (range: 3–38 months) months, respectively. During follow-up, 31 (19.5%) patients died, 26 (16.4%) of them due to UTUC; 28 (17.6%) patients experienced bladder recurrence.

**Figure 1:**
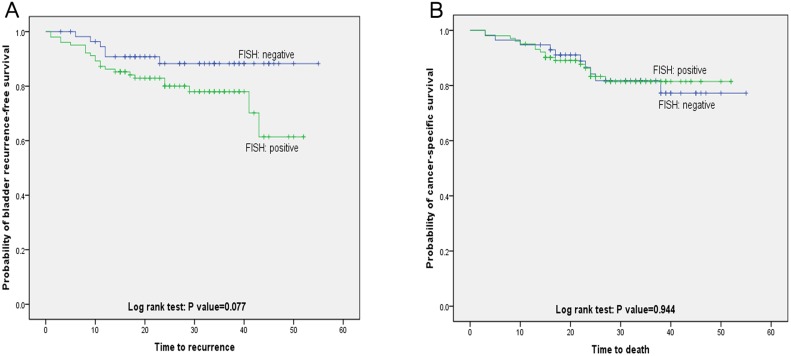
Bladder recurrence-free survival and cancer specific-free survival stratified by FISH result (**A**) Kaplan-Meier plot of recurrence-free survival curves stratified by positive and negative FISH (*p* = 0.077). (**B**) Kaplan-Meier plot of cancer specific-free survival curves stratified by positive and negative FISH (*p* = 0.944).

**Figure 2:**
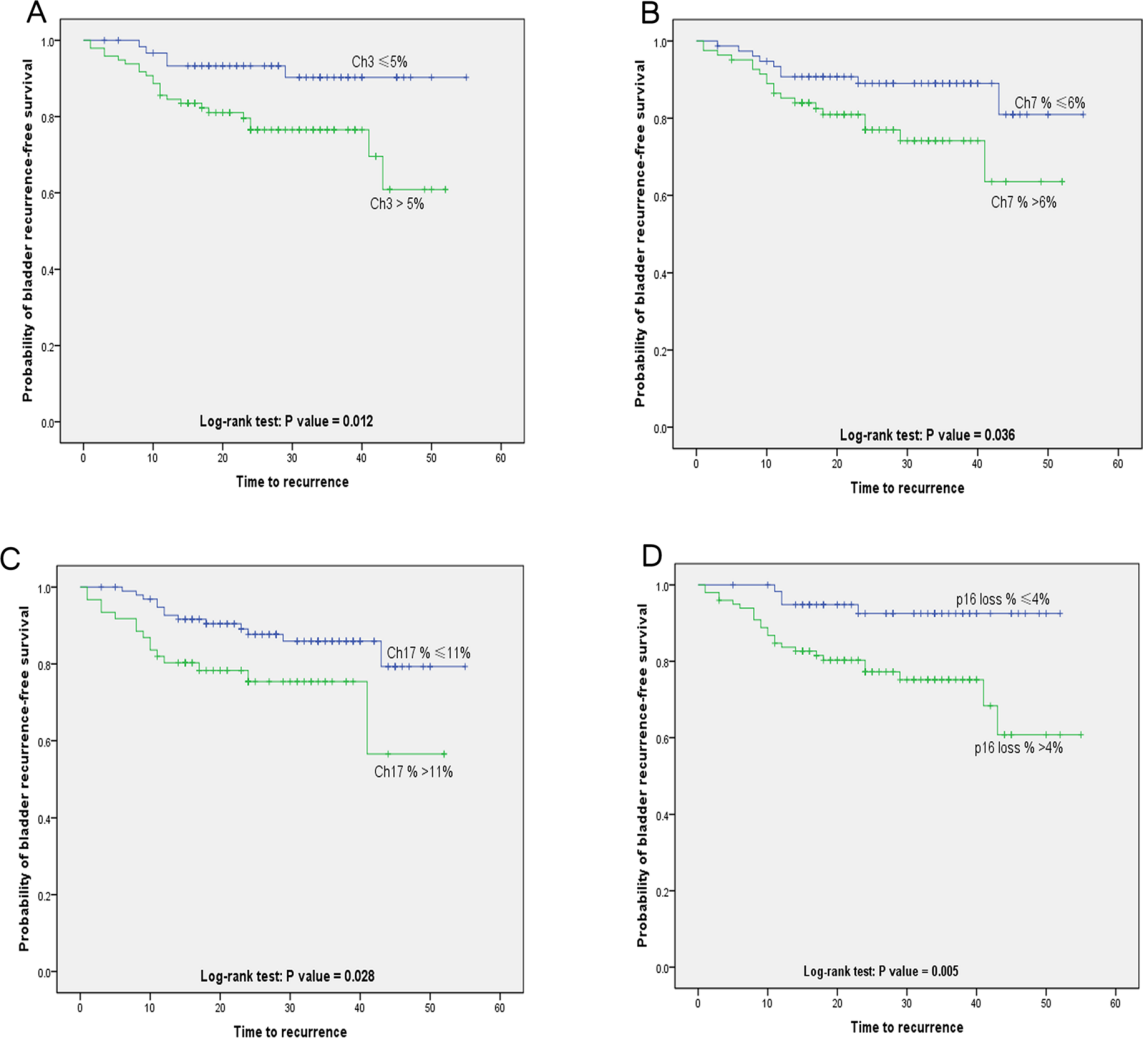
Kaplan-Meier plot of Bladder recurrence-free survival stratified by the percentage of copy number aberrations of (**A)** chromosomes 3 (*p* = 0.012), (**B**) chromosomes 7 (*p* = 0.036), (**C**) chromosomes 17 (*p* = 0.028) and (**D**) p16 loss (*p* = 0.005).

Variables associated with CSS and BRFS on univariate and multivariate analysis are shown in Table 4. The results of FISH analysis showed that 57 of 159 patients were negative, of 6 patients had histologically verified bladder recurrence; 102 patients were positive, of 22 had bladder recurrence. Kaplan–Meier analysis showed that patients with positive FISH result were more likely to present bladder recurrence (*p* = 0.077, Figure 1A). However, positive FISH was not related to cancer specific-free survival (CSS) (*p* = 0.944, Figure 1B). Tumor multifocality, the percentage of abnormal chromosome 3 > 5%, chromosome 7 > 6%, chromosome 17 > 11% and deletion of p16 > 4% were prognostic risk factors for BRFS in univariate analysis (Figure 2A–2D). Tumor multifocality (HR = 3.487, 95%CI: 1.605–7.576, *p* = 0.002) and p16 loss > 4% (HR = 3.766, 95%CI: 1.303–10.884, *p* = 0.014) were prognostic factors for BRFS in multivariate analysis. For CSS analysis, the percentage of abnormal chromosomes 3, 7, 17 and deletion of p16 were not prognostic factors in univariate analysis. Higher stage (HR = 6.087, 95%CI: 2.766–13.395, *p* < 0.001) and tumor size > 3 cm (HR = 2.425, 95%CI: 1.077–5.458, *p* = 0.015) were independent prognostic factors for CSS in multivariate analysis.

**Table 4:** Univariate and multivariate analysis of CSS and BRFS

Variable	CSS				BRFS			
	Univariate		Multivariate		Univariate		Multivariate	
	HR(95%CI)	*P*	HR(95%CI)	*P*	HR(95%CI)	*P*	HR(95%CI)	*P*
Gender Male Female	2.131 (0.949–4.782)	0.067			1.823 (0.852–3.901)	0.122		
Age	0.999 (0.960–1.038)	0.943			0.975 (0.942–1.009)	0.146		
Hydronephrosis Present Absent	1.490 (0.512–4.336)	0.464			1.199 (0.411–3.497)	0.740		
Tumor location Pelvis Ureter Both	1.709 (0.906–3.224)	0.098			1.297 (0.690–2.439)	0.420		
Tumor stage T1&T2 T3&T4	6.507 (2.962–14.296)	**< 0.001**	6.087 (2.766–13.395)	< 0.001	1.572 (0.691–3.575)	0.281		
Tumor grade High Low	2.898 (1.260–6.667)	**0.012**			1.224 (0.583–2.573)	0.593		
Lymph node status cN0 or pN0 N+	3.773 (1.125–12.648)	**0.031**			0.811 (0.110–5.969)	0.837		
LVI Presence Absence	3.867 (1.674–8.936)	**0.002**			2.216 (0.894–5.488)	0.086		
Tumor size ≤3cm >3cm	2.730 (1.214–6.137)	**0.015**	2.425 (1.077–5.458)	0.032	0.915 (0.428–1.957)	0.819		
Multifocal Yes No	1.889 (0.758–4.709)	0.172			3.808 (1.756–8.258)	**0.001**	3.487 (1.605–7.576)	0.002
Ch3 abnormal ≤ 5% > 5%	0.883 (0.405–1.924)	0.754			3.204 (1.217–8.343)	**0.018**		
Ch7 abnormal ≤ 6% > 6%	0.762 (0.349–1.662)	0.494			2.283 (1.029–5.065)	**0.042**		
Ch17 abnormal ≤ 11% > 11%	1.386 (0.634–3.030)	0.413			2.260 (1.070–4.774)	**0.033**		
p16 loss ≤ 4% > 4%	2.288 (0.918–5.706)	0.076			4.065 (1.410–11.718)	**0.009**	3.766 (1.303–10.884)	0.014

CSS: Cancer Specific-free Survival; BRFS: Bladder Recurrence-free Survival; LVI: lymphovascular invasion; CH: chromosome.

## DISCUSSION

UTUC is a comparatively rare cancer. The common diagnosis methods mainly included computed tomography/magnetic resonance imaging, cystoscopy, urinary cytology and diagnostic ureteroscopy. About 15–25% of bladder lesions are muscle invasive, while 60% of UTUC are invasive at initial diagnosis as well as patients with UTUC had comparatively poor prognosis [12]. The prognostic factors for UTUC included acknowledged tumor stage and grade, lymphovascular invasion status, lymph node involvement, tumor size, hydronephrosis, tumor multifocality and necrosis [12]. In addition, European Association of Urology Guidelines reviewed several literatures and reported that four nomograms were currently available to predict survival rates post-operatively, based on standard pathological features [13–16]. Several studies had demonstrated that ureteroscopy increased the risk of bladder recurrence [17, 18], but urinary markers which could predict tumor recurrence were relatively few. Nobuyuki Tanaka et al. [19] retrospectively collected 474 patients with non-metastatic UTUC and found post-operative positive urine cytology was correlated with the occurrence rate of intravesical relapse after RNU. Kobayashi et al. [20] reported that urine cytology with positive result was a independent prognostic marker for intravesical relapse using a cohort of 252 UTUC patients. Our previous study [8] suggested that positive FISH result of urine implied invasive UTUC (sensitivity for muscle-invasive and high grade UTUC, 71.70% and 76.47%, respectively) and this result gave aid to the clinical urological surgeon to select an appropriate operation method. However, the connection between FISH results and survival or progression of UTUC patients was unclear. Based on above study, we established this research to assess the influence of positive FISH on oncological outcomes in UTUC.

In this research, the results showed that patients with positive FISH were more likely to experience bladder recurrence. Casey Seideman et al. [11] reported that FISH result could accurately predict intravesical recurrence for those patients with bladder cancer. Jared Whitson et al. [6] reported that patients with high-risk bladder deseases were more likely to relapse when they had positive FISH results. Massimo Maffezzini et al. [21] analyzed 126 patients diagnosed with non-muscle invasive bladder cancer (NMIBC) and observed that FISH-positive results was highly predictive of relapse. Our result determined the prognostic significance of FISH for predicting oncologic outcome. Recent fundamental research suggested that the luminal seeding and implantation hypothesis played a major role in bladder recurrence [22, 23]. This hypothesis mainly asserted that the multifocal development including simultaneous and metachronous tumor was caused by the planting or intraepithelial diffusion tumor cells. Since positive FISH result were associated with higher stage and grade tumor which presented more aggressive, the patients with positive FISH result possibly had higher degrees of intravesical exposure. Therefore, it was reasonable that positive FISH patients tend to relapse in bladder.

In this cohort, we were the first to define the cutoff value of preoperative FISH test probe as a predictive indicator of bladder recurrence. In univariate analysis, tumor multifocality, the percentage of abnormal chromosome 3 > 5%, chromosome 7 > 6%, chromosome 17 > 11% and deletion of p16 > 4% were correlated with BRFS. In multivariate analysis, only tumor multifocality and p16 loss > 4% were prognostic factors for BRFS. The 9p21 which contained p16/CDKN2 and p14ARF site located in chromosome 9 short arm and mainly participated in regulating cell circle and cell death [24, 25]. The loss of 9p21 leads to the p16 gene non-functional, and then disorder cell cycle resulting in disease relapse [26]. Hideyasu Matsuyama et al. [10] identified 118 bladder wash samples of patients with NMIBC and found that 9p21 loss larger than12% was a predictive factor for relapse. Zellweger and his research team [27] found that the loss of p16 was expressively related to bladder relapse. Kawauchi and his colleagues [28] confirmed that a 9p21 index was an independent prognostic factor for bladder recurrence in patients with urothelial carcinoma. Although the p16 site was one of the most common genetic mutations in urothelial carcinoma [29], but our results were paradoxical. Traditionally, a tube of urine sample was diagnosed FISH with positive result in patients with urothelial tumor if at least 12 cells (12%) with homozygous p16 deletions were identified, but we set 4% as cutoff values of p16 loss based on AUC from ROC for recurrence. One possible explanation for our finding may be that p16 genetic aberrations of Chinese UTUC patients were relatively low compared with western country, hence the possibility of p16 loss we could detect from voided urine was comparatively low (median of p16: 7%). Consequently, p16 was a special characteristic for Chinese UTUC patients and its clinical significance should be taken into consideration.

Our study also has some limitations.. First of all, the present study was confined due to its retrospective features and considerably small sample size. In addition, the FISH approach might present with interobserver evaluation diversity and diverse specimens quality. Despite these limitations, this is the first report so far of the capacity of FISH to assess its predictive value for UTUC bladder recurrence. Although a multicenter perspective cohort study with longer follow-up period should be performed to identify these research results, our study group found that preoperative FISH probe data could not only be used as a diagnostic tool and predict UTUC tumor malignant behavior, but also could be used as a prognostic tool.

## MATERIALS AND METHODS

### Patient selection

A total of 232 consecutive patients who underwent surgeries for UTUC in the Urology Department, Peking University First Hospital between January 2012 and May 2016 were enrolled in this study. Seventy-three were subsequently removed from the analysis: 22 patients with previous or concomitant bladder tumors; 36 patients failed to follow up; 15 patients without information of FISH probe. Clinicopathologic data for the remaining 159 patients were retrospectively analyzed. None of the patients had received neoadjuvant chemotherapy. All patients provided written informed consent.

### FISH, treatment strategy and pathological evaluation

Voided urine specimens from the 159 UTUC patients were analyzed using UroVysion FISH kit; labeled probes specific for chromosomes 3, 7, and 17, and the p16 (9p21) genes were used to assess prognostic value. About 45 ml urine of each patient was collected using a 50 ml sterile centrifuge tube from 8:00 to 11:00 in the morning. The FISH assays were ordered based on provider's protocol. Diagnosis criteria for positive FISH was described as previous study [8]. All UTUC patients who underwent conservative surgery or RNU were regularly followed according to institutional practice. In this cohort, one hundred and forty-two patients underwent RNU with an ipsilateral bladder cuff excision; the remaining 17 patients underwent conservative surgery, including ureteroureterostomy and complete distal ureterectomy and neocystostomy. All pathological specimens were reexamined by a dedicated genitourinary pathologist to confirm the original diagnosis. Tumor stage, grade, multifocality, tumor size and status of regional lymph nodes were described as previous study [8].

### Follow-up

Follow-up including laboratory data, chest X-ray, urinary ultrasonography, computed tomography/magnetic resonance imaging and cystoscopic evaluation of the urinary bladder should be made every 3 months for the first 2 years, every 6 months until year 5, then annually. The co-primary endpoints of the study were bladder recurrence and cancer-specific death. Bladder recurrence was defined as finding bladder tumors at cystoscopic evaluation and the patients were performed transurethral resection of bladder tumor or radical cystectomy thereafter. Bladder recurrence-free survival (BRFS) and cancer-specific survival (CSS) were calculated from the date of surgery to the date of bladder recurrence and cancer specific death.

### Statistical analysis

We used Mann-Whitney *U* and chi-squared tests to compare continuous and categorical variables, respectively. Binary logistic regression was used to evaluate preoperative factors. The probability of survival was calculated by the Kaplan–Meier method, with statistical differences evaluated by the log-rank test. Variables influencing BRFS and CSS were compared using Cox proportional hazards regression models. Variables with a *p* < 0.05 in univariate analysis were also assessed in multivariate analysis. All statistical analyses were performed using the IBM Statistical Package for Social Sciences (SPSS) version 22.0. For all statistical tests, two-sided *p* < 0.05 was considered to indicate statistical significance.
